# Supporting frontline staff in frontotemporal degeneration research

**DOI:** 10.3389/fneur.2026.1906002

**Published:** 2026-09-11

**Authors:** Shana G. Dodge, Danelle J. Carter, Dahlia Kamel, Angela Lunde, Quinn Hlava, Kevin Nelson, Penny A. Dacks

**Affiliations:** 1 The Association for Frontotemporal Degeneration, King of Prussia, PA, United States; 2 Department of Neurology, University of Colorado Anschutz, Aurora, CO, United States; 3 Department of Neurology, Frontotemporal Degeneration Center, Perelman School of Medicine, University of Pennsylvania, Philadelphia, PA, United States; 4 Department of Neurology, Mayo Clinic Rochester, Rochester, MN, United States; 5 FTD Disorders Registry, King of Prussia, PA, United States

**Keywords:** clinical research coordinators, frontotemporal degeneration, neurodegenerative disease research, research staff survey, research workforce training

## Abstract

**Introduction:**

Clinical Research Coordinators (CRCs) serve as the frontline staff for research participants in studies on frontotemporal degeneration (FTD), directly influencing participant experience. The training adequacy, job satisfaction, and retention of FTD CRCs have not been systematically examined.

**Methods:**

The Association for Frontotemporal Degeneration (AFTD) administered an anonymous cross-sectional online survey to CRCs affiliated with research networks across the USA and Canada.

**Results:**

Thirty-seven CRCs responded. Most had more than 3 years of experience, yet 62% reported encountering situations for which they felt inadequately prepared. Information on genetics, symptom management, and brain donation were identified as critical training gaps. Retention was linked to competitive pay and clear career advancement opportunities.

**Discussion:**

The findings highlight a critical need for formalized, disease-specific onboarding and emotional support systems tailored to CRCs that engage with FTD patients and families. AFTD is developing a modular training curriculum to standardize CRC competency.

## 1 Introduction

Frontotemporal degeneration (FTD), also referred to as frontotemporal dementia, is an umbrella term for a group of relatively rare, progressive, terminal neurodegenerative conditions impacting the frontal and temporal lobes, that results in changes in behavior and personality, communication, and motor functioning. FTD phenotypes include behavioral variant FTD (bvFTD), primary progressive aphasia (PPA), progressive supranuclear palsy (PSP), corticobasal degeneration (CBD), and FTD with amyotrophic lateral sclerosis (FTD-ALS). FTD disorders typically begin in midlife and differ in their clinical presentation, underlying pathology, and causes; however, subtype diagnosis is generally based on clinical symptoms (1).

While there are currently no approved symptomatic or disease-modifying therapies for FTD, active research is expanding across interventional and observational settings. The success of these efforts, however, hinges on the Clinical Research Coordinator (CRC). Often called study coordinators, CRCs play a significant participant-facing role and are responsible for the day-to-day activities of a research program, including frequent and meaningful interactions with participants and their families (2).

From a participant perspective, the CRC is often the most salient face of the research team. They are frequently the first professionals a person diagnosed and their family encounter who possess genuine knowledge of FTD. Their role spans the entire study lifecycle, from screening and consent to navigating complex site visits. Consequently, the empathy and competency of the CRC are critical to ensuring a positive experience for families navigating a devastating diagnosis. Trusted relationships with research staff and meaningful engagement have been identified as key factors in participant retention (3).

There is no single standardized entry pathway for the CRC role, and the level of education and training required for CRCs to reach competency varies considerably, which challenges conventional workforce development approaches. The training and credentialing process for CRCs often begins with foundational education—typically in life sciences or nursing—and mandatory instruction in Good Clinical Practice (GCP) and human subject protection, structured around the Joint Task Force for Clinical Trial Competency framework (4). New coordinators build expertise through supervised, hands-on site management, participant recruitment, and regulatory administration, supplemented by ongoing peer-led professional development (5, 6). After accumulating 2–3 years of full-time operational experience, CRCs sometimes pursue voluntary, globally recognized professional certifications—such as ACRP’s Certified Clinical Research Coordinator (CCRC^®^) or SOCRA’s Certified Clinical Research Professional (CCRP^®^), which require passing a standardized competency exam and maintaining active credentials through biennial continuing education (7).

The stability of CRC staffing is a critical determinant of clinical trial efficiency, financial sustainability, and scientific validity (8–10). It is also critical to recruitment and retention, both of which are a primary challenge in FTD trials that usually seek to recruit small subsets of diagnosed people with specific genetic variant (11). Typical recruitment and retention challenges are more pronounced in rare neurodegenerative diseases due to small eligible populations, higher per-participant costs, geographic dispersion, and progressive cognitive and behavioral decline, which can prevent continued participation regardless of motivation, and a heavy reliance on care partners for participation (12–14).

Stable CRC staffing mitigates these risks by improving study continuity, reducing protocol inconsistency, and supporting participant confidence (15, 16). The turnover rate of CRCs, however, is high because the role is often considered a temporary springboard to pursue additional education or career paths (15). The broad literature indicates that CRC retention is significantly driven by both competitive compensation and the quality of the Principal Investigator (PI) relationship (15, 16). Ultimately, institutional investment in market-based salary adjustments, combined with a PI’s hands-on engagement in training and professional development, remains the most effective strategy for stabilizing this workforce (17, 18).

Further, the literature increasingly identifies a therapeutic competency gap, in which CRCs receive adequate training in administrative tasks but lack sufficient depth of knowledge in the disease areas they support. Research indicates that protocol complexity is a top barrier to trial success, often because staff lack the specialized medical knowledge required to explain advanced mechanisms of action to participants (19, 20). This lack of disease-specific investment is directly linked to higher turnover and recruitment failures, as coordinators who do not feel scientifically confident are less effective at consenting complex patients and identifying disease-related adverse events (5, 15, 20).

In neurodegenerative disease research—where coordinators must navigate cognitively impaired participants alongside study partners, longitudinal retention across multi-year protocols, genetic testing and counseling for at-risk families, brain donation discussions, and disease-specific symptom and progression knowledge—no published survey has directly characterized what coordinators report needing to learn. This absence is especially pronounced in FTD, where the supporting research infrastructure remains comparatively nascent: consortium-based training and standardized site-personnel resources are well established in Huntington’s disease (e.g., HDSA’s longstanding genetic testing protocol and Enroll-HD’s centralized certification of site staff) and in Alzheimer’s disease (e.g., the Alzheimer’s Disease Research Center network and purpose-built genetic disclosure processes for prevention trials), but FTD lacks an equivalent, mature scaffold (21, 22).

In 2024, the Association for Frontotemporal Degeneration (AFTD), a patient advocacy organization dedicated to FTD disorders, conducted a closed-door industry-sponsored advisory panel to explore issues related to FTD, including barriers to clinical research participation. Through these, as well as *ad hoc* conversations with people with lived experience of FTD and with clinical research professionals, the critical role of CRCs was highlighted.

“It’s almost like rolling out the red carpet. If you want people to participate, you need to make it easier in all aspects of their journey.”

“That site went through 5 study coordinators…the study coordinators all sounded like they were about 14 years old. They may have been quite competent, but their communication skills were not.”

## 2 Materials and methods

In 2025, AFTD launched a cross-sectional survey of CRCs to better understand their experiences, gaps in training, and factors related to job satisfaction. The anonymous, online survey was created by the FTD Disorders Registry and distributed to all study coordinators working on the ARTFL-LEFFTDS Longitudinal Frontotemporal Lobar Degeneration (ALLFTD) Program, a large, North American, multi-site natural history study focuses on FTD; shared through AFTD social media channels; and sent to CRCs who had recently interacted with the AFTD. The survey was designed based on topic areas that arose during the 2024 AFTD industry-sponsored advisory panel as well as follow up interviews with community members, clinical research coordinators, and principal investigators. The draft survey was reviewed and revised based on feedback from several clinical research coordinators, including those that manage others; the FTD Disorders Registry Manager; the FTD Disorders Registry President and AFTD Chief Scientific Officer; the FTD Disorders Registry ALLFTD Liaison; and ALLFTD Program Managers.

The survey was built on WordPress using WPEngine. The survey was approved by the North Star Review Board and was open from October 8, 2025 through November 24, 2025. Data are stored on a secure, cloud-based platform with role-based access controls and password-protected user authentication. Access to data is limited to authorized study personnel and system administrators whose responsibilities require such access. Data are maintained within a secure environment that incorporates industry-standard technical and administrative safeguards. This survey did not collect personally identifiable information, and all data are maintained in a de-identified format. Regular security monitoring and access management procedures help protect the confidentiality, integrity, and availability of data. Voluntary informed consent was obtained from all human subjects.

The survey (Supplementary material) included 16 questions and three optional demographic questions. Only respondents who selected one of the following options on an initial screener proceeded to the full survey: I work as a clinical research coordinator in FTD research, I used to work as an FTD clinical research coordinator, or I supervise FTD clinical research coordinators. Questions were both multiple choice as well as open-text and were designed to gather information on CRCs level of experience and the type of research they work in, situations in which they did not feel adequately prepared, areas in which they would have liked additional training, where they go for emotional and logistical support, career plans, and what they like and do not like about their role.

Multiple choice responses were analyzed using Microsoft Excel pivot tables. Open-text responses were initially analyzed for themes using Claude Opus 4.8. These responses and themes were then verified manually to ensure they were accurately reflected and no themes omitted.

## 3 Results

**Figure 1:**
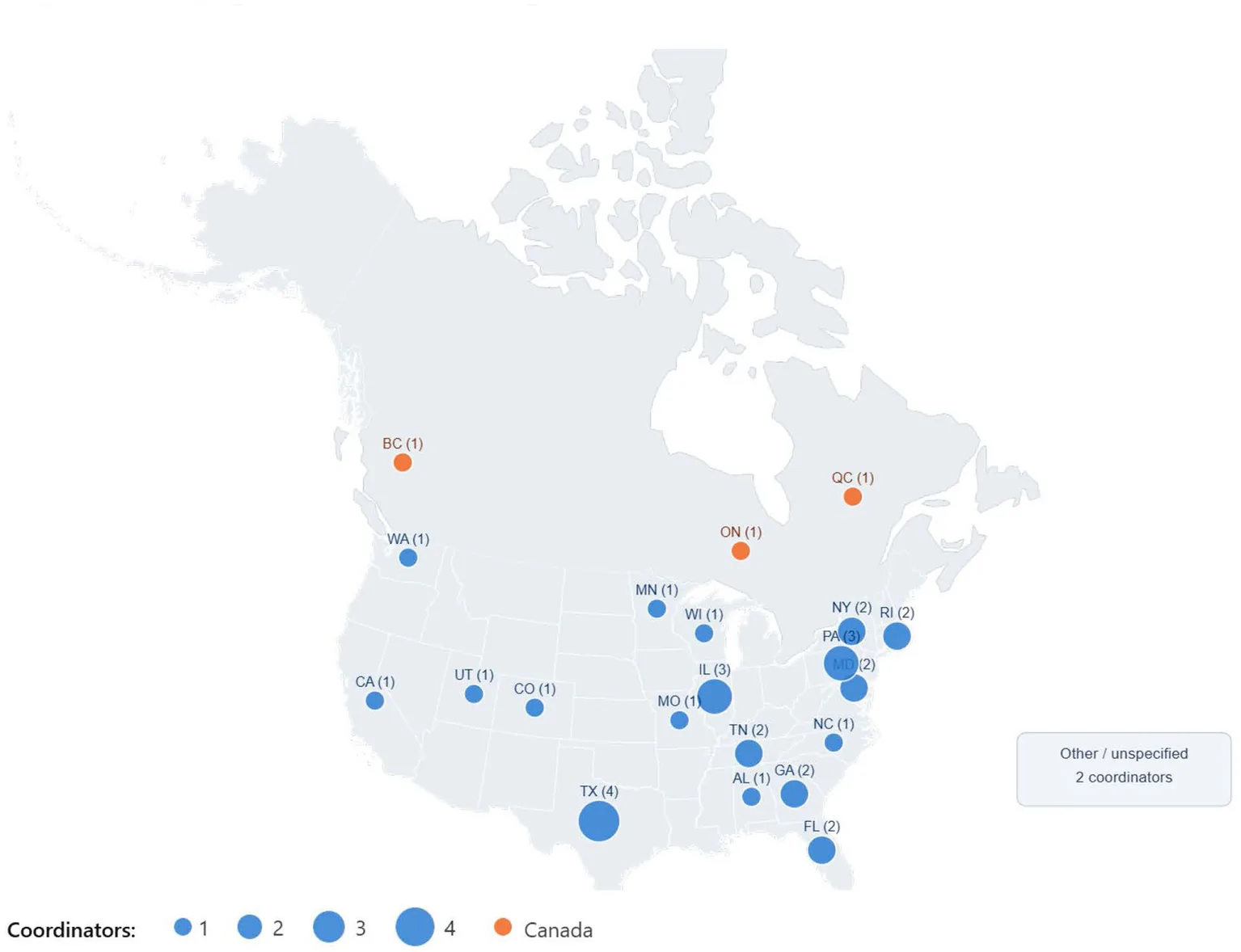
Geographic distribution of respondents.

Thirty-seven CRCs completed the survey. As the survey was shared on social media, it is not possible to calculate the exact response rate, however, it was shared with at least 64 ALLFTD CRCs, meaning a response rate of 58% or less, which is relatively consistent with other survey studies (23). Twenty of the 27 (74%) ALLFTD sites were represented. The majority of respondents (79%) identify as female and white (53%) (see Table 1). Respondents were affiliated with 27 institutions across the USA and Canada (see Figure 1).

**Table 1:** Demographics of survey respondents.

Demographic variable	Optional response
Age in years [M (SD)], (*N* = 27)	31.07 (8.26)
Gender identity (*N* = 34)	Number (percent)
Genderqueer, nonbinary, or genderfluid	2 (6%)
Man	1 (3%)
Woman	27 (79%)
Prefer not to respond	4 (12%)
Race or ethnicity (*N* = 36)	Number (percent)
American Indian or Alaska Native	0 (0%)
Asian	3 (8%)
Black or African American	2 (6%)
Hispanic or Latino/a/é	5 (14%)
Middle Eastern or North African	2 (6%)
Native Hawaiian or Other Pacific Islander	1 (3%)
White	19 (53%)
Prefer not to respond	4 (11%)

All demographic information was optional.

Table 2 summarizes respondent’s description of their current role. Most had been in FTD research for more than 2 years, with 46% having worked in FTD research for 3–4 years, 24% for more than 5 years, 24% for 1–2 years, and 5% for less than 1 year. Most (58%) did not manage anyone else, while 31% managed other research coordinators. Respondents varied in terms of the percentage of time spent interacting directly with participants with 39% endorsing 25–49% of their time in direct participant contact, 22% 1–24% of their time, 28% 50–74% of their time, 8% 75–100% of their time and 3% (a single respondent) 0% of their time.

**Table 2:** Results of the survey relating to current research role.

How long have you worked in FTD research?	*N* = 37
Less than one year	2 (5%)
1–2 years	9 (24%)
3–4 years	17 (46%)
5 + years	9 (24%)

What is your current role?	*N* = 36
I am a research coordinator and do not manage anyone else	21 (58%)
I am a research coordinator and manage other research coordinators	11 (31%)
Other*	4 (11%)

Approximately what percentage of your time is spent interacting directly, either in person or virtually, with research participants?	*N* = 36
0%	1 (3%)
1–24%	8 (22%)
25–49%	14 (39%)
50–74%	10 (28%)
75–100%	3 (8%)

*Other: clinical research coordinator supervisor, program manager, patient coordinator.

Table 3 summarizes the type of research in which CRCs participate. Respondents worked in both observational and interventional studies with 44% working in both, 44% working exclusively in observational/natural history studies, and 11% solely in interventional/clinical trial research. Respondents also varied with respect to participant population; 53% worked primarily in FTD research in addition to other diseases, 33% primarily in other diseases but on FTD studies as well, and 14% exclusively in FTD research. Respondents described a wide range of research functions, with the vast majority (92%) implementing a full research role (e.g., recruitment, enrollment, running visits and visit planning, communication with participants, monitoring, budget) as well as data management (e.g., entering data, queries, data quality).

**Table 3:** Results of the survey relating to research type.

The type of research I work in is	*N* = 36
Only observational/natural history studies	16 (44%)
Only interventional/clinical trials	4 (11%)
Both observational/natural history studies *and* interventional/clinical trials	16 (44%)

I work	*N* = 36
Primarily in FTD research, but in other disease areas as well	19 (53%)
Primarily in non-FTD research (for example, Alzheimer’s disease), but on some FTD studies as well	12 (33%)
Exclusively in FTD research	5 (14%)

The type of work I do includes (select all)	*N* = 37
Full research role: recruitment, enrollment, running visits and visit planning, communication with participants, monitoring, budget, etc.	34 (92%)
Data management: entering data, queries, data quality	34 (92%)
Regulatory (for example, IRB submissions, compliance, protocol review, monitoring)	25 (68%)
Specific research role (for example, genetics, brain donations, neuropsychological testing, laboratory)	20 (54%)
Finance/budgets	13 (35%)
Other*	3 (8%)

*Other: strategic planning of projects within the program and maintaining clinical research operations; team leadership; managing medications, patient education.

Most (49%) respondents were not familiar with FTD prior to their FTD CRC role while 38% had professional experience working in related conditions. Table 4 summarizes past FTD experience.

**Table 4:** Results of the survey relating to familiarity with FTD.

Before you worked in this role, were you familiar with FTD? [select all]	*N* = 37
I was not familiar with FTD	18 (49%)
I had professional experience working in related conditions, such as Alzheimer’s disease or ALS	14 (38%)
I had professional experience in research, but outside of the neurodegenerative field	7 (19%)
I had professional experience with FTD, such as working in FTD care or learning about it in school	5 (14%)
I had personal experience with FTD	4 (11%)

Respondents were asked to identify situations for which they did not feel adequately prepared. These included answering questions about FTD genetics (41%), answering participants/study partner’s questions about how to manage FTD symptoms (38%), answering participants’ questions about FTD (30%), answering participants’ questions about brain donation/autopsy (24%), and managing participants’ behaviors (24%). Thirty-eight percent reported that they feel they have been adequately trained (see Table 5).

**Table 5:** Results of the survey relating to situations for which they were not prepared.

Have you found yourself in a situation in your role for which you did not feel adequately trained? (select all that apply)	*N* = 37
No	14 (38%)
Yes, I did not feel fully prepared to answer participants’ questions about FTD genetics	15 (41%)
Yes, I did not feel fully prepared to answer participants/study partner’s questions about how to manage FTD symptoms	14 (38%)
Yes, I did not feel fully prepared to answer participants’ questions about FTD	11 (30%)
Yes, I did not feel fully prepared to answer participants’ questions about brain donation/autopsy	9 (24%)
Yes, I did not feel fully prepared to manage participants’ behaviors (e.g., aggressive behavior)	9 (24%)
Yes, I had difficulty answering questions about medications	7 (19%)
Yes, I had difficulty communicating with someone because of their language symptoms	6 (16%)
Yes, I did not receive training on basic research requirements, such as regulatory, ethics, and documentation requirements	4 (11%)
Yes, I did not feel fully prepared to answer participants’ questions about clinical trials	4 (11%)
Yes, I had difficulty managing a study partner’s behavior or emotions	4 (11%)
Yes, I had difficulty managing someone’s motor/movement symptoms (for example, helping them navigate the visit while in a wheelchair)	3 (8%)
Yes, I did not feel fully prepared to manage the logistics of study visits (for example, having the correct equipment for study visits, completing research database steps, handling technological support questions)	2 (5%)
Other*	4 (11%)

*Other: I did not feel fully prepared to answer participants’ questions about the research protocol or consent form; to appropriately administer neuropsychological or cognitive testing; to address the concerns and/or support participants whose culture or background was different than my own, or different from what I had expected.

Likewise, participants were asked what areas in which they would have liked additional training, these included FTD genetics (51%), the basics of FTD (49%), brain donation/autopsy (43%), resources available to people with FTD and their families (43%), and what it is like to live with FTD or care for someone with FTD (30%). Sixteen percent reported no additional training is needed. These results are summarized in Table 6.

**Table 6:** Results of the survey relating to perceived training needs.

Either now, or at the time I started my role, I would have liked additional training in (select all)	*N* = 37
No additional training needed	6 (16%)
FTD genetics	19 (51%)
The basics of FTD	18 (49%)
Brain donation/autopsy	16 (43%)
Resources available to people with FTD and their families (for example, support groups, legal resources)	16 (43%)
What it is like to live with FTD or care for someone with FTD	11 (30%)
Appropriately consenting a legally authorized representative (LAR)	10 (27%)
How to have difficult or emotionally-charged conversations with participants (for example, dealing with positive genetic testing results)	10 (27%)
How to work with participants with language difficulties	9 (24%)
General research training and education (for example, regulatory, ethics, documentation, protocol review, how to stay audit read), risk reporting for adverse events	8 (22%)
How to manage difficult behaviors	8 (22%)
Consenting participants with FTD	7 (19%)
How to address the concerns and/or support participants of different ethnocultural backgrounds (for example, the needs of people from rural communities, different ethnocultural norms in behavior, or when English is not their first language)	7 (19%)
How to pursue a career in FTD research	7 (19%)
Research finances (for example, budgeting, invoicing)	6 (16%)
The FTD Disorders Registry	6 (16%)
The logistics of FTD research study visits	6 (16%)
How to work with participants with motor/movement difficulties	6 (16%)
How to read FTD research articles	5 (14%)
Neuropsychological or cognitive testing	5 (14%)
Typical study procedures (for example, MRI, lumbar punctures)	5 (14%)
How to manage my own feelings related to working families with FTD (for example, setting personal boundaries, dealing with grief)	5 (14%)
Opportunities to observe/shadow other staff conducting study visits	4 (11%)
Observational research and the basics of clinical trials	4 (11%)
Practicing elements of study visits (for example, role playing, mock study visits)	4 (11%)
The relationship between natural history studies and clinical trials	3 (8%)
The differences between research and clinical care	3 (8%)

Table 7 summarizes questions related to perceived support and professional goals. The majority of respondents reported they find logistical and emotional support from their PI (81%) and other research management (58%). Others reported finding support from their fellow research coordinators (53%) and other FTD clinical staff (50%). Only 6% (2 respondents) noted they do not have support in the logistics of, or emotions related to working with people with FTD. While the majority (64%) reported that they plan to stay working in the FTD field, 31% noted that they are unsure. For those who said they do not plan on staying in the FTD field (5%), or are unsure, they would be more willing to stay in the FTD field if they saw room for growth at their institution (46%), there was better pay (38%), they better understood career opportunities in FTD research or clinical care (31%), or they felt more emotionally supported at work (23%).

**Table 7:** Results of the survey relating to support and professional goals.

When I need support in the logistics of or emotions related to working with people with FTD, I can find support [select all]	*N* = 36
From my PI	29 (81%)
From other research management	21 (58%)
From other FTD research coordinators	19 (53%)
From other FTD clinical staff (for example, nurses, neurologists)	18 (50%)
From research coordinators working in other disease areas	14 (39%)
Outside of work (for example, from my friends or family)	13 (26%)
I do not have this kind of support	2 (6%)
Social workers at my site	1 (3%)

I plan to stay working in the FTD field	*N* = 36
Yes	23 (64%)
No	2 (6%)
Unsure	11 (31%)

‘I would be more willing to stay in the FTD field if [select all]*	*N* = 13
I saw room for growth at my institution	6 (46%)
There was better pay	5 (38%)
I better understood career opportunities in FTD research or clinical care	4 (31%)
I felt more emotionally supported in my work	3 (23%)
There were more FTD studies ongoing at my site	2 (15%)
I had more opportunities to understand the science of FTD (for example, attend scientific conferences)	2 (15%)
I felt more technically supported in my work (for example, had more training on how to administer FTD clinical tools)	2 (15%)

*Asked of respondents who responded No or Unsure to “I plan to stay working in the FTD field”.

In an open text prompt, respondents were asked what they like most about their jobs. CRCs consistently describe their work as deeply meaningful and purpose-driven, feeling like they are making a difference in people’s lives, contributing to cutting-edge or important research, and helping families find purpose and hope through research participation. Respondents also emphasized human connection: building long-term, longitudinal relationships with participants; being present for both people diagnosed and care partners; and creating a sense of family, trust, and safety during an extremely difficult time.

CRCs framed themselves as witnesses to participants’ lived experiences, emotional supports during difficult disease journeys, and translators between science and human experience. CRCs also reported how engaged they are with care partners, not just people diagnosed and often act as advocates, bridging families and the healthcare/research system and providing education, resources, and emotional support.

“Being able to provide families with a unique research experience that makes them feel like family during an extremely difficult time.”

“Patient interaction and providing support to the caregivers even if just by way of letting them vent.”

“The most rewarding part of my role is working with patients and learning how they are affected by FTD. Their personal stories are inspiring and are a constant reminder of why we do this important research/who we are helping.”

“I like interacting with and supporting my research participants. I’ve grown to know and care for them so it’s nice to be able to support them when possible and learn from them.”

CRCs also reported enjoying the diversity of tasks and intellectual stimulation which give them a wide range of responsibilities (regulatory, grants, manuscripts, participant contact). The dynamic, non-repetitive nature of the work, along with opportunities to learn about disease, research, and people, contributes to job satisfaction. They also appreciate the blend of clinical care and research, working longitudinally, and helping participants understand their condition while balancing participant-facing responsibilities with administrative and regulatory work. Several comments point to the importance of the organizational environment, noting pride in leadership and program structure, strong team cohesion and shared values, opportunities to teach and mentor new or early-career research professionals, and a commitment to quality, preparation, and compassion.

“The variety of activities that I am involved with, from regulatory affairs to grant preparation, manuscript writing, and participant and family contact.”

“This role also involves a wide variety of tasks which keeps things interesting.”

“The impact it makes on research participants and the field.”

“Teaching young, new clinical research professional about this field and this disease is an honor.”

**“**For our team, I feel confident in saying that all of us go beyond just doing our jobs but to be there for our patients and their families.”

In an open text prompt, respondents were asked what they like least about their jobs. CRCs reported structural, emotional, and disease-specific challenges. A dominant theme was insufficient staffing relative to workload including understaffing and high staff turnover, being responsible for “all the logistics” alone, wearing multiple hats; being “thrown into the deep end,” long hours just to accomplish core responsibilities, and feeling undercompensated for the scope of responsibility.

“Understaffing and staff turnover.”

CRCs also reported time scarcity and competing priorities, with difficulty completing all aspects of study visits in limited time, multitasking and prioritization challenges, managing documentation and regulatory requirements alongside participant-facing care, scheduling conflicts with participant, care partners, and clinicians, and managing multiple commitments simultaneously.

“Scheduling with patients and clinicians is challenging. It is also difficult to manage multiple commitments.”

“Coordinators are expected to maintain a lot of moving parts. I think it can be challenging for coordinators to understand the full scope of the work, maintain the regulatory/administrative aspects of the job, and be fully present in visits to provide quality care.”

CRCs highlighted the high logistical burden of running studies, including complicated study visit logistics, difficulty with recruitment and follow-through in longitudinal studies, managing unexpected situations during visits, navigating institutional constraints and restrictions, and maintaining operational standards while also training staff.

Financial instability appears as a recurring challenge with uncertainty around funding for salaries, lack of communication around study pauses or delays due to funding, lack of funding for additional staff, and institutional limitations shaping what can or cannot be done.

“Figuring out funding to support my own and staff’s salaries.”

Another theme was the disease-specific emotional toll of the work, including the loss of participants and associated grief; watching the decline and death of participants including cases involving younger participants with children; supporting families over the course of disease progression; the difficulty of processing these emotional experiences within or outside of work; and the need to maintain professional boundaries while forming close relationships.

“Seeing our patients decline fast and passing within a short time, especially when children are involved.”

“The most difficult part of my job is losing my participants. We’ve had a fair amount of participants pass in the past year and I try to manage my grief around that and build healthy boundaries but it’s hard when I get to know them and their families so well.”

Respondents clearly identified FTD as uniquely challenging due to the inherent behavioral and personality changes; managing wandering, impulsivity, and eating behaviors; communicating with participants with language symptoms related to PPA; re-explaining information due to cognitive impairment; and navigating challenging care partner dynamics. Recruitment appeared as both a logistical and emotional burden, an already persistent challenge amplified by disease severity and family burden.

“In terms of working with FTD, cases [are] often more challenging than other dementias like Alzheimer’s due to their significant behavioral and personality changes. However, my understanding of FTD has help me be better equipped mentally to handle difficult cases of FTD during their research visits.”

CRCs struggled with what they can and cannot provide, noting an inability to answer all participant and caregiver questions; delivering difficult news (study delays, disease progression); navigating participant and clinician personalities; and needing clearer triage pathways for mental health and social services.

“The most difficult part is not being able to answer all of the questions asked to us by the patients. Some of the questions are areas in which we cannot help them specifically as a research coordinator so I think it’s important to have a “triage” at sites for mental health, at home services, and other areas that patients/caregivers may need extra assistance with.”

Finally, in an open text response, CRCs were asked what they would want sites to know to better support them in their role. CRCs reported wanting more disease-specific onboarding; more support for themselves and their participants regarding the feasibility of long, visits; more resources on navigating difficult study visits; and a career path beyond the “stepping-stone.”

“I think having more online resources like conversation guides, helpful tips, etc. for research professionals would be helpful. Even better if these guides were pulled from care partners and patient’s feedback on their experiences with researchers during a study visit.”

“Just because the targeted population is known from a clinic perspective does not mean they are capable, or willing, to sit through multiple hours of testing…very few of our site’s participants over the years have been local, so it takes a lot of planning, time and resources to make these visits happen and rarely are they ever fully ‘completed’.”

“Different training courses that could be integrated in the regular hiring process.”

“While compiling multiple facets of research within one study is helpful to the researcher, it is not always what’s best for the patient. Asking affected individuals to do several difficult tasks within a visit for minimal return has proven to decrease involvement at our site.”

“There is a need for more conversation on career opportunities beyond the option to continue higher education.”

“Training on the spectrum of FTD disorders is so valuable.”

## 4 Discussion

The FTD CRC role is operationally complex, covering a broad range of responsibilities. CRCs reported an inherent tension between administrative demands and quality participant engagement as well as role overload and structural vulnerability in coordinator positions. The majority of survey respondents manage the “full research role” (recruitment through monitoring) as well as data management. In addition, most CRCs surveyed spend 25–74% of their time in direct contact with participants, requiring high emotional intelligence and clinical management skills. Despite additional challenges unique to working with people facing FTD, only 14% work exclusively in FTD; the vast majority juggle FTD with other neurodegenerative diseases like Alzheimer’s.

Despite the majority being in their roles for more than 3 years, a large portion of CRCs feel under-equipped to handle the specific complexities of FTD; 62% of respondents reported facing situations for which they were not adequately trained and nearly half (49%) entered their roles with no prior familiarity with FTD. Many pointed to wanting training in areas that may not be fully the responsibility of the CRC themselves, but that arise during study visits. There was a clear call for therapeutic area training to be formalized and integrated directly into the hiring process with CRCs explicitly asking for enhanced training on FTD, genetics, and brain donation as well as help with soft skills, such as navigating difficult behaviors and sensitive conversations. Some CRCs reported having to learn on the job, calling for a more structured hiring and onboarding curriculum. CRCs also noted sometimes being unclear on what they can and cannot do, reflecting a degree of role ambiguity and emotional strain tied to scope-of-practice limits.

CRCs further expressed a sense of burden, both for themselves and participants, when balancing protocol design and participant capacity. They feel that researchers often overlook the physical and cognitive exhaustion that multiple hours of testing can cause for affected individuals. The feedback highlights a disconnect between protocol design and the practical realities of travel logistics, care partner burden, and visit feasibility, noting that visits are “rarely ever fully completed” due to these constraints. Notably, 11% reported having personal experience with FTD, which can lend them a deeper, more empathic understanding of the disease while also potentially introducing additional emotional burden.

The survey results also suggest a dedicated workforce, but one that faces a bottleneck regarding long-term career growth. The majority (70%) have been in FTD research for 3 or more years, indicating a solid foundation of expertise rather than high immediate turnover. For those hesitant to stay in FTD, the primary drivers are a lack of room for growth at their institution and adequate pay. This suggests that while CRCs find the work meaningful, they may leave the field if they cannot find a clear career trajectory or competitive compensation. CRCs rely heavily on their immediate academic hierarchy for support; the PI is the most cited source of support, but peer support, internal management and other FTD CRCs, are also vital. Only 3% (one person) reported using social workers at their site for support, which is a potentially missed opportunity given the high emotional burden of the disease. There is a clear desire to see clinical research as a long-term profession rather than just a precursor to graduate studies. Additional support may include PIs and other research leaders having conversations with CRCs about career opportunities within the research infrastructure itself.

The survey, deployed in fall 2025, coincided with a period of notable instability in the federal research funding environment. An Association of American Medical Colleges analysis found that NIH committed nearly $5 billion less in research grants to U. S. institutions over the prior year than in the year before, the result of disruptions and slowdowns in the grant-awarding process (24, 25). Against this backdrop, it is understandable that coordinators would identify budgetary and financing uncertainty as a salient concern and a barrier to remaining in the field, given the direct link between funding stability and the security of coordinator positions.

There are limitations of this study. Notably, the pool of CRCs surveyed may not be representative of the larger community of CRCs. They were predominantly female and white and the sample size was modest at 37 completed responses. Most CRCs had been in their role for several years and were primarily located at major medical centers. The dissemination methodology also does not allow for a thorough analyses of response rates. Further, the small sample size did not allow for comparative analyses, nor did it collect detailed information on current training protocols to explore, for example, differences between CRCs based on their site location, availability of resources (e.g., an on-site genetic counselor), educational background, site training CRC protocol, specific research focus (e.g., different diagnostic FTD types; those who work exclusively in FTD research compared to those who work primarily in other disease spaces). Future studies should consider how these and other variables may impact a CRC’s perceived and actual degree of proficiency.

Institutional and PI recognition of the key role of CRCs within FTD research could result in direct improvements in study participant recruitment, retention, and well-being, as well as study protocol completion. Despite respondents being relatively more senior and invested in completing this survey, the CRCs surveyed are calling for more comprehensive training, increased emotional and logistical support, and clearer professional development guidance. AFTD is currently working in conjunction with ALLFTD and other research stakeholders to design a virtual, modular FTD CRC training to help streamline access to the specialized skills required for FTD research. Supporting CRCs not only creates a pipeline of bright, enthusiastic, empathic FTD researchers, but also enhances the participant experience, improves retention and increases the likelihood that this unique and crucial population will continue to participate in research.

## Data Availability

The raw data supporting the conclusions of this article will be made available by the authors, without undue reservation.
