# Supplementary material for: Supporting frontline staff in frontotemporal degeneration research
Source: Front Neurol. 2026 Sep 11;17:1906002. doi: 10.3389/fneur.2026.1906002 (PMC13612334; doi:10.3389/fneur.2026.1906002)
Supplement: Supplementary file 1 [file Data_Sheet_1.PDF]

CRC Training Survey

The Association for Frontotemporal Degeneration (AFTD) is conducting a survey to better understand the critical role of frontotemporal degeneration (FTD) **clinical research coordinators** and how they can be best supported in their work. All questions are optional and you can stop taking this survey at any time. All responses will be pooled and nothing will be traced back to you personally.

The results of this survey will help us develop training and support materials for clinical research coordinators.

Definitions:

FTD = frontotemporal degeneration, including behavioral variant FTD (bvFTD), primary progression aphasia (PPA), FTD with amyotrophic lateral sclerosis (ALS) or motor neuron disease (MND), corticobasal degeneration or corticobasal syndrome (CBD/CBS), progression supranuclear palsy (PSP)

Participants = anyone who participates in FTD research, including persons diagnosed, care partners, study partners, biological family members, people at genetic risk for FTD

Select the category that best describes you: [select one]

- I work as a clinical research coordinator in FTD research
- I supervise FTD clinical research coordinators
- I used to work as an FTD clinical research coordinator
- I have never worked as a clinical research coordinator in FTD research [end survey]

Demographics

1. Where do you work? [pick one]

    - Brown University
    - Case Western Reserve Medical Center
    - Columbia University
    - Emory University
    - Indiana University
    - Johns Hopkins University
    - Massachusetts General Hospital
    - Mayo Clinic - Jacksonville
    - Mayo Clinic - Rochester
    - Mount Sinai
    - Nantz National Alzheimer Center, Houston

- National Institute of Health (NIH)/National Institute of Neurological Disorders and Stroke (NINDS)
- Northwestern University
- University of Alabama, Birmingham
- University of British Columbia
- University of California Los Angeles
- University of California San Diego
- University of California San Francisco
- University of Colorado Denver
- University of Michigan
- University of North Carolina Chapel Hill
- University of Pennsylvania
- University of Texas Health Science Center at San Antonio
- University of Toronto
- University of Washington
- Vanderbilt University
- Washington University in St. Louis
- Other: [open text]

2. How long have you worked in FTD research (in your current position or in other roles)? [pick one]
    - Less than one year
    - 1-2 years
    - 3-4 years
    - 5+ years

3. What is your current role? [pick one]
    - I am a research coordinator and do not manage anyone else
    - I am a research coordinator and manage other research coordinators
    - Other [open text]

4. Approximately what percentage of your time is spent interacting directly, either in person or virtually, with research participants?
    - 0%
    - 1-24%
    - 25-49%
    - 50-74%
    - 75-100%

5. Before you worked in this role, were you familiar with FTD? [select all]
    - I had professional experience with FTD, such as working in FTD care or learning about it in school

- I had professional experience working in related conditions, such as Alzheimer's disease or ALS
- I had professional experience in research, but outside of the neurodegenerative field
- I had personal experience with FTD
- I was not familiar with FTD

6. The type of research I work in is [select all]
    - Observational/natural history studies (for example, ALLFTD)
    - Interventional/clinical trials
    - Other [open text]

7. I work [pick one]
    - Exclusively in FTD research
    - Primarily in FTD research, but in other disease areas as well
    - Primarily in non-FTD research (for example, Alzheimer's disease), but on some FTD studies as well

8. The type of work I do includes [select all]

    - Full research role: recruitment, enrollment, running visits (all parts), visit closeout, monitoring, budget, etc
    - Data management: entering data, queries, data quality
    - Regulatory (for example, IRB submissions, compliance, protocol review, monitoring)
    - Finance/budgets
    - Specific research role (for example, genetics, brain donations, neuropsychological testing, laboratory)
    - Other: [open]

9. Have you found yourself in a situation in your role for which you did not feel adequately trained? [select all that apply]
    - Yes, I didn't feel fully prepared to answer participants' questions about FTD
    - Yes, I did not receive training on basic research requirements, such as regulatory, ethics, and documentation requirements
    - Yes, I didn't feel fully prepared to answer participants' questions about the research protocol or consent form
    - Yes, I didn't feel fully prepared to answer participants' questions about clinical trials
    - Yes, I didn't feel fully prepared to answer participants' questions about FTD genetics
    - Yes, I didn't feel fully prepared to answer participants' questions about brain donation/autopsy

- Yes, I didn't feel fully prepared to manage participants' behaviors (for example, aggressive behavior)
- Yes, I had difficulty communicating with someone because of their language symptoms
- Yes, I had difficulty managing someone's motor/movement symptoms (for example, helping them navigate the visit while in a wheelchair)
- Yes, I didn't feel fully prepared to answer participants/study partner's questions about how to manage FTD symptoms
- Yes, I had difficulty answering questions about medications
- Yes, I had difficulty managing a study partner's behavior or emotions
- Yes, I didn't feel fully prepared to appropriately administer neuropsychological or cognitive testing
- Yes, I didn't feel fully prepared to manage the logistics of study visits (for example, having the correct equipment for study visits, completing research database steps, handling technological support questions)
- Yes, I didn't feel fully prepared to address the concerns and/or support participants whose culture or background was different than my own, or different from what I had expected (for example, participants from rural communities where travel requirements or technology access may be an issue, participants with different ethnocultural norms or language than my own, or participants with disabilities)
- Other [open text]
- No

10. Either now, or at the time I started my role, I would have liked additional training in [select all]
    - The basics of FTD
    - Observational research and the basics of clinical trials
    - Practicing elements of study visits (for example, role playing, mock study visits)
    - Neuropsychological or cognitive testing
      General research training and education (for example, regulatory, ethics, documentation, protocol review, how to stay audit read), risk reporting for adverse events)
    - Typical study procedures (for example, MRI, lumbar punctures)
    - Consenting participants with FTD
    - Research finances (for example, budgeting, invoicing)
    - Appropriately consenting a legally authorized representative (LAR)
    - Opportunities to observe/shadow other staff conducting study visits
    - The logistics of FTD research study visits
    - The relationship between natural history studies and clinical trials
    - FTD genetics

- Brain donation/autopsy
- How to manage difficult behaviors
- How to work with participants with language difficulties
- How to work with participants with motor/movement difficulties
- Resources available to people with FTD and their families (for example, support groups, legal resources)
- How to manage my own feelings related to working families with FTD (for example, setting personal boundaries, dealing with grief)
- How to have difficult or emotionally-charged conversations with participants (for example, dealing with positive genetic testing results)
- How to pursue a career in FTD research
- How to read FTD research articles
- The differences between research and clinical care
- What it is like to live with FTD or care for someone with FTD
- The FTD Disorders Registry
- How to address the concerns and/or support participants of different ethnocultural backgrounds (for example, the needs of people from rural communities, different ethnocultural norms in behavior, or when English is not their first language)
- Other [open text]
- No additional training needed

11. When I need support in the logistics of or emotions related to working with people with FTD, I can find support [select all]
    - From my PI
    - From other research management
    - From other FTD research coordinators
    - From other FTD clinical staff (for example, nurses, neurologists)
    - From research coordinators working in other disease areas
    - Outside of work (for example, from my friends or family)
    - I don't have this kind of support
    - Other [open text]

12. I plan to stay working in the FTD field [pick one]
    - Yes
    - No
    - Unsure

13. If selected No/Unsure in #11: I would be more willing to stay in the FTD field if [select all]
    - I saw room for growth at my institution
    - I felt more emotionally supported in my work

- I felt more technically supported in my work (for example, had more training on how to administer FTD clinical tools)
- There were more FTD studies ongoing at my site
- There was better pay
- I better understood career opportunities in FTD research or clinical care
- I had more opportunities to understand the science of FTD (for example, attend scientific conferences)
- Other [open text]

14. What do you like most about your job? [open text]

15. What is most difficult about your job? [open text]

16. Is there anything else you would want study sites to know to better support you in your role as an FTD research coordinator? [open text]

Optional demographic information:

Age: [open text]

Which of the following best defines your current gender identity? Select all that apply:

- Genderqueer, nonbinary, or genderfluid
- Man
- Woman
- Prefer to self-describe [open text]
- Prefer not to respond

Which of the following best defines your race or ethnicity? Select all that apply:

- American Indian or Alaska Native
- Asian
- Black or African American
- Hispanic or Latino/a/é
- Middle Eastern or North African
- Native Hawaiian or Other Pacific Islander
- White
- Prefer to self-describe [open text]
- Prefer not to respond
